# Side errors in neurosurgery and human factors training

**DOI:** 10.1007/s00701-014-2326-z

**Published:** 2015-01-15

**Authors:** Patrick Mitchell, Trevor Dale

**Affiliations:** 1 Department of Neurosurgery, Royal Victoria Infirmary, Newcastle upon Tyne, NE1 4LP UK; 2 Atrainability, Maraquita, 42 Horsham Road, Cranleigh, Surrey GU6 8DU UK

**Keywords:** Human factors, Crew resource management, Side errors, Surgery, Briefing, Safety

## Abstract

**Background:**

We previously reported on a series of side errors in cranial neurosurgery that occurred around the UK before the year 2006. That survey was prompted by a cluster of six cranial and spinal side errors that occurred in the neurosurgery department in Newcastle upon Tyne during the year 2006. The report was part of our investigation into the problem and how to solve it.

**Methods:**

A human factors training programme was run in the department in response to a further side error. All 125 members of the neurosurgical theatre staff attended 1 of 5 training days. Fifteen days of professional observation and coaching were held within the theatre suite. Time between errors was recorded. The success or otherwise of human factors measures such as checking and briefing was observed.

**Results:**

A side checking system was adopted and became universal. Pre-list briefing meetings were adopted and quickly became widely used but took several years to become universal. Post-list debriefing meetings were introduced but were not widely adopted and quickly fell out of use. Mean time between side errors was 2 months pre-intervention, 18 months after introducing a standardised checking system and 82 error free months had passed since the human factors training programme.

**Conclusions:**

Side errors in neurosurgery can be reduced by a combination of systematic checking and education. We suspect that education is useful in reducing error rates from low to very low but, as is generally true of human factor interventions, the evidence for this is soft.

## Introduction

Operating on the wrong side is a special case of wrong site surgery that is poorly tolerated by the general public because of its easily understandable nature. The resulting sound bites attract media coverage and public anxiety, often out of proportion to any harm done, and lead to emotional or political rather than scientific reactions that tend to be ineffective at preventing recurrence.

Prior to the mid-1990s, wrong side surgery received little scientific attention though was a recurring cause of litigation. In 1997 the American Academy of Orthopaedic Surgeons (AAOS) formed a task force on wrong site surgery that measured the incidence of such errors and began a “Sign Your Site” campaign to promote preoperative site marking. This was followed by similar campaigns by the Joint Commission on Accreditation of Healthcare Organizations, US Veteran’s Health Administration, Canadian Orthopaedic and the North American Spine Society Associations [1]. These highlighted pre-operative site marking as a mainstay of error prevention. The value of pre-operative site marking varies between surgical disciplines and is mixed in neurosurgery. Many neurosurgical operations, even when sided, are done through incisions that are not side specific. The anterior cervical spine is usually approached from the right side whichever side is the target of the operation because it is easier for right-handed surgeons. Similar situations are posterior approaches to the spine, bifrontal and midline posterior fossa craniotomies and trans-sphenoidal surgery. Among them these approaches account for a majority of operations done in most neurosurgery units. Pre-operative site marking has become the norm in UK neurosurgery over the past 10 years but these limitations mean it is not a complete solution to the specific problem of side errors.

We have previously published a series of eight side errors that occurred in cranial neurosurgical operations in the British Isles between 1999 and 2006 [2], undoubtedly a small proportion of total errors. Six side errors occurred in Newcastle during 2006 including spinal and cranial operations and action was taken to prevent recurrence. This involved a universal “knife check” consisting of a verbal challenge response check with at least two members of staff participating to confirm patient identity, site and side immediately before the scalpel was handed over to commence an operation. This reduced the error rate from a high base by about nine fold but a further error occurred. In this article we examine this error and action taken to further reduce the error rate.

## Materials and methods

Ethical approval was not required for this study. Our previous paper was restricted to the analysis of cranial surgery side errors. In this report our investigation was focussed on all side errors, be they spinal or cranial and including operations that were done on the wrong side through a midline incision such as lumbar microdiscectomies and decompressive foramenotomies or facetectomies.

### Ascertainment of errors

We ascertained all errors that were reported via the critical incident reporting system that was in use at the time. This system detected two out of a total of six errors that we became aware of during the calendar year 2006. The other errors were identified on post-operative imaging in three cases and on repeat surgery in one case. As with our previous report, the nature of these errors and the requirement for confidentiality put significant limitations on what details can be given.

In response to this series of errors we conducted the survey published previously [2]. From this survey we designed a checking system that would have trapped all errors that we were aware of, had it been in use. This has become known as a knife check. The check is a verbal challenge response check between at least two members of staff with no paper record being made. It is made immediately before the scalpel or other instrument is handed to the surgeon to commence the procedure, hence the term “knife check”. It involves a check that information on imaging, the consent form and the patient agree in name, date of birth, and operative site and side. In the first 3 years we used date of birth only to supplement identification but since 2009 we have also used the hospital number. This check forms part or our implementation of the Time Out of the WHO operative check list.

#### Implementation

A specific Monday was set to implement the knife check. The intended implementation date was announced verbally and with posters in theatres 1 week before the date. On the day of implementation the head of neurosurgery (PM) spent the day in theatre without having a list. He went round all theatres reminding and ensuring that the knife check was carried out as intended. This role was taken on by other surgeons for the remaining days of the first week. All nursing staff, anaesthetic staff and ODPs were encouraged to initiate such a knife check if it had not been initiated by the surgeon. Scrub staff were instructed not to hand over the scalpel until the check had been completed to their satisfaction. It was intended to audit compliance with the knife check after 1 month but in the event the impression was that compliance had reached 100 % after 2 weeks. After the check had been in place for 2 weeks, individual staff members were identified and asked to assess compliance in theatres over a 2-day period. This assessment showed the compliance had reached 100 %. A further assessment was planned for 6 months later. This was originally going to be conducted over 2 days but by the end of the first day it was clear that compliance was 100 % and the check had been embedded in the culture of the theatres.

#### A further error

No further errors were identified via any means including critical incident reporting, revision surgery or post-operative imaging during the 12 months following the introduction of the knife check. A further error however did occur 18 months after the knife check had been introduced.

An adult male patient was to have a posterior fossa craniotomy and removal of a left-sided meningioma. The operation was being done by a registrar. The consultant in charge of the case was involved in an emergency operation in another theatre. He left the positioning to the registrar while he went to assess progress with the other operation. He returned to theatre to check on positioning. The head had been positioned as for a right-sided operation but the consultant did not notice the error and was not present when the knife check was done.

The wound was prepared and draped on the right-hand side. The knife check was completed as normal. The anaesthetist, scrub nurse and operating surgeon conferred on the name, date of birth, and site and side of the surgery. They agreed that it was to be a left operation and also agreed that it was the left side that was prepared. Surgery continued accordingly. A right-sided lateral posterior fossa incision was made and muscle dissection began. The consultant returned to theatre before the skull had been perforated, checked the scans and recognised the error. He halted the operation, pointed out the error and conducted a hot debrief into how the error had arisen.

From the debrief it emerged that both the scrub nurse and surgeon had left-right dyspraxia and normally had to stop and think to check sides rather than knowing left from right intuitively. This was compounded by the patient being prone. It also emerged that the anaesthetist had checked the name and date of birth of the patient from the consent form but had not personally checked the operative site because he had been located at the foot of the patient and the operative site was obscured even when he was standing. It also emerged that leading questions had been used in the knife check. The significance of this was not appreciated at the time but several months later it emerged that much of the department had fallen into the habit of using leading questions for the knife check.

Following this incident and its investigation a meeting was held with the aim of improving the checking system to prevent a recurrence. The knife check itself had been fairly straightforward to design and implement but there was no agreement on how to improve it further. It was agreed to seek expert help.

With this in mind we contacted the human factors department at Cranfield University. We were put in contact with a team of past and present BA pilots who were involved in introducing human factors lessons learned in aviation into health care (Atrainability). This commercial company provides training in human factors. The North East Strategic Health Authority Patient Safety Group was approached and funded a training programme. All employees involved in neurosurgical theatres (125 employees in total) were given the training that consisted of a 1-day course. Five such courses were held over a period of 3 months. The courses were held during working hours and a total of four full day operating sessions were cancelled to accommodate them.

Together with the taught course, theatre coaching was also used. For 15 days over a 3-month period (July–September 2009) Atrainability experts observed theatre behaviour and advised as appropriate. The total cost of the intervention was £45,000 + 4 theatre days. One of the authors (PM) spent 3 days of purely passive observation of theatre behaviour during October 2014 to assess long-term compliance.

## Results

Course participants were polled on the utility of the course within a week of their completion. The results of a total of 86 respondents are shown in Fig. 2.

### Theatre observation

Several issues emerged from the theatre observation exercise of 2009:

#### Knife check

The check was being done universally at the beginning of the observation period but was variously referred to as a “knife check” or “time out”. The check was being done reliably by more than one person with a challenge-response format but the initial challenge frequently involved leading questions such as *Is it the left side?* rather than *Which side is it?* A consequence of the training courses was that the significance of leading questions was realised. It was realised that this had contributed to the last error and furthermore that it was a quite widespread practice. The knife check protocol was altered to specifically avoid the use of leading questions.

#### Briefing

Pre-list briefing was done variably. Briefing rates increased from around 10 % of lists at the beginning of the observation period to around 90 % at the end. The benefits and skills of briefing had been a part of the 1-day course. Briefing had not been used at all before the courses were run and steadily increased as more staff members had been through the course.

#### Debriefing

Debriefing was also covered in the course, which advocated formal debriefing meetings at the end of operating lists. These were tried. They had never been done prior to the intervention. For an initial period of a few weeks during and after the courses such meetings were held for up to 50 % of lists.

### Medium- and long-term results: 3 months to 5 years after the training programme

#### Knife check

The knife check remained universal.

Leading questions occasionally recur but are corrected because of heightened staff awareness of the issue.

#### Briefing

The rate of briefing remained at around 90 % of lists until 2014. This was because a small minority of the consultant surgeons used an alternative format to pre-list briefing for team communication. This changed in the wake of a near miss involving confusion over the order of cases on a list. During October 2014, 15 all-day operating lists were observed. All had briefings at 0830 a.m.

#### Debriefing

By 6 months after the intervention, formal debriefing meetings at the end of lists had fallen out of use completely. Staff were asked the reasons for this failure. Two issues dominated responses:

At the end of an operating list, different team members’ tasks end at different times so that there is no time for a meeting that is convenient to everyone.

Debriefing has less immediacy as it is perceived to be concerned with long-term process improvement rather than the conduct of a specific list and therefore has lower priority than briefing.

In response to the failure of formal debriefing we have encouraged a system of hot debriefing, in which issues, good or bad, are “debriefed” as soon as is practicable after they arise. This system is not so amenable to uptake measurement so data are lacking.

#### Error rates

**Fig. 1:**
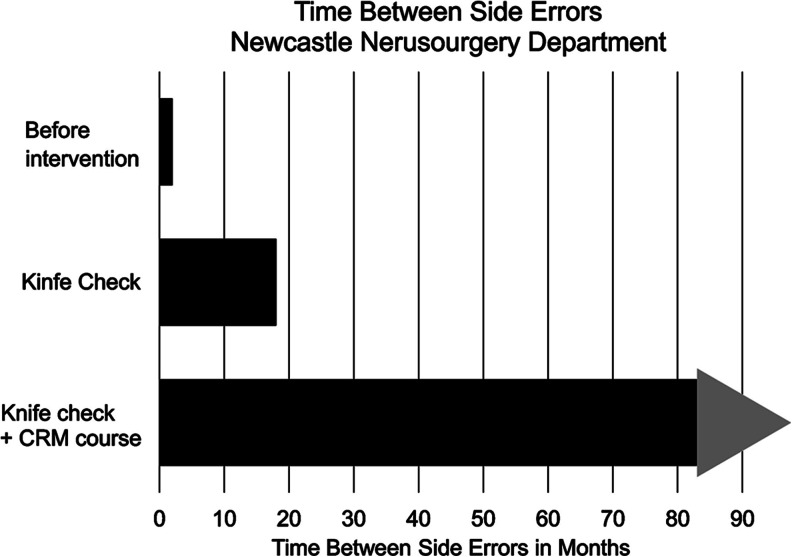
Time between errors before intervention (2006), after the knife check was introduced and after the training programme (Crew Resource Management or CRM course as it was then called)

No further side errors have been recorded. At the time of writing the side error-free period is 82 months during which time 25,812 procedures were performed in operating theatres by the department. We do not have complete data electronically on sidedness of operations so a sample of 100 of these were selected at random. Eighty-two of these were sided and 18 un-sided or midline. We therefore estimate a side error-free run of approximately 21,000 sided operations. Time between errors is shown in Fig. 1.

#### Follow-on training

**Fig. 2:**
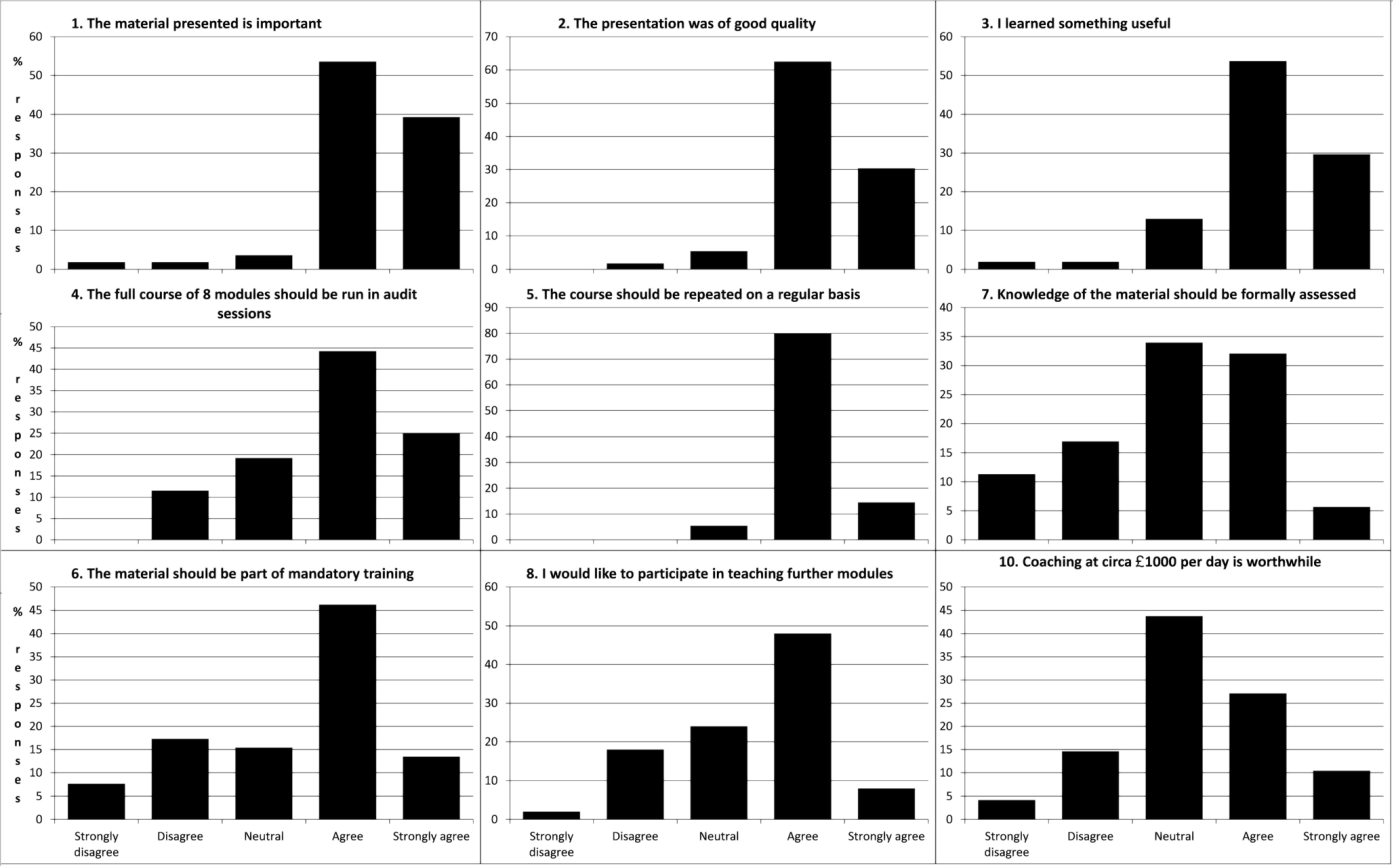
Results of course participants’ feedback. Eighty-six respondents out of 125 total participants

It was anticipated from the beginning that the single intensive training programme would be effective for a period of time but would become less effective as staff moved or memories faded. It was therefore planned to implement regular update training. At the time there was no training programme available at a manageable cost. A successful grant application was made to the Health Foundation to develop such a course and this course was published in 2013 [3, 4]. Implementation in the Newcastle neurosurgical department has begun but a formalised system of training of all the staff is still pending (Fig. 2).

## Discussion

The complexity of causative factors in human errors means that we can never be certain whether the introduction of the course was wholly responsible for the final diminution in the side error rate. Furthermore it is expected that eventually another error will be made and our goal of error monitoring is to increase time between errors rather than eliminate errors entirely.

Although our programme of human factors training was triggered by the issue of side errors, the subject’s scope is far wider than that with much greater potential benefits in terms of patient safety, staff satisfaction, staff stress and work efficiency. A number of factors limited our ability to measure these changes. In particular fairly shortly after the course was completed the neurosurgery department moved to a new hospital with inevitable staff and environmental changes. Isolating effects of the course from the effects of the move is therefore difficult.

Our experience with briefing was that it gained rapid, wide and durable acceptance. Our experience with formalised debriefing was the opposite. Even while pressure was being applied to hold debriefing, penetrance into practice did not exceed 50 % of lists and once pressure was removed it rapidly and completely fell out of use. There are a number of reasons for this including there being no time that is convenient to everyone and the lack of immediate relevance of debriefing and locating debriefing at the end of the working day brings the potential for it to delay departure from the workplace.

Given our own experience it is perhaps not surprising that research into the long-term effects of debriefing in surgery is lacking. In theory briefing is about short-term operational efficiency whereas debriefing is about long-term process improvement. Debriefing is of higher strategic but lower operational importance than briefing. Debriefing is supposed to provide a negative feedback process control loop that promotes practices that work and abandons those that do not. These potential benefits justify further research. We are encouraging the hot debriefing system described but penetrance and effectiveness assessment is pending. Other units have experimented with a debriefing book where issues are entered as they arise to be discussed at specific times though we have not tried this.

## Conclusions

Human errors in surgery are manageable. Where error rates are unexpectedly high, simple measures can be effective, but reducing rates from low to very low requires increased focus and effort. It is uncertain whether this effort is merited on the grounds of one narrow kind of error; however we consider the benefits are far broader than the error that triggered the intervention in our case and recommend increased awareness and training in human factors to all health care teams.
